# Tolerability and Adverse Effects in a Specialized Heart Failure Guideline‐Directed Medical Therapy Optimization Program

**DOI:** 10.1002/clc.70179

**Published:** 2025-07-15

**Authors:** Claudia Mae Velasco, Gladys Baksh, Michele Haydo, Heather Reesor, John Boehmer, Omaima Ali

**Affiliations:** ^1^ Penn State College of Medicine Hershey Pennsylvania USA; ^2^ Division of Cardiology, Heart & Vascular Institute Penn State Hershey Medical Center Hershey Pennsylvania USA

**Keywords:** adverse drug reactions, guideline‐directed medical therapy, heart failure with reduced ejection fraction, tolerance

## Abstract

**Background:**

Utilization of heart failure (HF) guideline‐directed medical therapy (GDMT) to target doses is suboptimal, with studies citing adverse effects (AEs), physiological factors, and therapeutic inertia as potential contributing factors. The objective of our study was to explore tolerability and GDMT titration‐limiting AEs in a specialized heart failure optimization program implemented at our institution.

**Methods:**

We studied the baseline characteristics of 254 patients who successfully completed our program and analyzed the frequency and severity of the four most common GDMT‐related AEs: hypotension, bradycardia, hyperkalemia, and renal dysfunction.

**Results:**

Patients who achieved target doses were younger, more likely to have nonischemic HF, less likely to have a recent HF‐related hospitalization, had less coronary artery disease, and were more likely to be obese. Multivariate analyses revealed significant associations between beta blocker suboptimal dosing (< 50% of target dose) and older age (odds ratio [OR]: 1.04; 95% confidence interval [CI]: 1.0–1.07; *p* = 0.031), presence of atrial fibrillation (OR: 2.57; 95% CI: 1.18–5.58; *p* = 0.017), and absence of hypertension (OR: 0.39; 95% CI: 0.17–0.89; *p* = 0.025). For angiotensin converting enzyme inhibitors/angiotensin II receptor blockers/angiotensin receptor neprilysin inhibitors, suboptimal dosing was associated with the presence of atrial fibrillation (OR: 2.08; 95% CI: 1.04–4.17; *p* = 0.039). Of the patients who completed the program, 59.1% encountered at least one AE that hindered the titration to target GDMT doses.

**Conclusion:**

Our findings highlight the complexities of GDMT optimization within a specialized program and the need for standardized definitions of GDMT‐related AEs and management strategies.

## 1 Introduction

Guideline‐directed medical therapy (GDMT) to target doses has led to greatly improved outcomes for patients with heart failure with reduced ejection fraction (HFrEF), yet its utilization is suboptimal in clinical practice, posing an increased mortality risk [1, 2, 3]. The CHAMP‐HF registry indicates that less than 75% of eligible patients receive recommended treatments with less than 2% having a documented absolute contraindication to any specific therapy [4]. When GDMT was prescribed, most received less than 50% of the target dose for beta‐blockers (BB) and angiotensin converting enzyme inhibitors/angiotensin II receptor blockers/angiotensin receptor neprilysin inhibitors (ACEi/ARB/ARNI) [4]. Longitudinal data show minimal dose increases over 12 months [5].

Several studies cite adverse effects (AEs) and limiting physiological factors as potential contributing factors to suboptimal use of GDMT. The most commonly reported AEs limiting GDMT titration are bradycardia, hypotension, hyperkalemia, and renal impairment [6]. Additionally, age plays a significant factor, with a Danish nationwide study demonstrating that older age is associated with lower proportions of daily target doses, reduced adherence, and a higher rate of discontinuation of ACEi/ARB and BB [7]. Reasons for lower adherence to GDMT in older adults include the increasing burden of comorbidities, fear of poor tolerability or adverse effects, and polypharmacy [8]. Despite these challenges and although guidelines do not recommend age‐related differences in medical approaches to HFrEF, older adults remain underrepresented in clinical trials [8].

An approach that has shown to improve the use and adherence to GDMT is the implementation of multidisciplinary HF care teams that incorporate remote management strategies [9, 10]. These programs are typically led by a nurse practitioner, pharmacist, or HF cardiologist, who employ telehealth methods to facilitate longitudinal surveillance of laboratory tests, blood pressure, and symptoms, as well as guidelines‐based medication adjustments. A remote multidisciplinary GDMT optimization program showed a significant increase in proportion of patients receiving at least half of the target dose for all GDMT categories in the intervention group, compared to the usual care group [10]. Despite the evidence supporting the potential of these programs to bridge the gap between guidelines and clinical practice, obstacles in uptitrating GDMT for certain patients remain.

This study aims to investigate tolerability and AEs that limit medication titration within a specialized GDMT optimization program at our institution.

## 2 Methods

### 2.1 Study Design

This was a single‐center, observational prospective and retrospective study conducted from September 2020 to July 2023. This study was approved by the institutional review board and deemed exempt under study 00021138.

### 2.2 Participants

We included 327 patients with HFrEF or heart failure with mid‐range ejection fraction (HFmrEF) enrolled in an outpatient HF‐GDMT optimization program at Penn State Health Milton S. Hershey Medical Center.

### 2.3 GDMT Program

The program was led by a HF nurse practitioner and clinical HF pharmacist [11]. Following referral, the GDMT titration period consisted of weekly to biweekly telehealth visits led by the pharmacist, during which participants self‐monitored their weight, blood pressure (BP), and heart rate (HR). These vital signs along with symptoms and laboratory values were reviewed to make appropriate adjustments to medication doses. Patients experiencing symptomatic hypotension and bradycardia had a threshold of systolic BP < 90 mmHg or HR < 55 beats/min. The use of sodium‐glucose cotransporter‐2 (SGLT2) inhibitors as part of the GDMT regimen became more standardized beginning in April 2022, following the 2022 AHA/ACC/HFSA updated guidelines on management of heart failure [12]. Medications were titrated sequentially, and the process continued until patients completed the GDMT program. Patients who could not achieve the target dose per guidelines after titration were considered to be on the maximum tolerated dose.

### 2.4 Statistical Analysis

Of the 327 patients enrolled, 254 patients successfully completed the program and were included in subsequent data analyses. We used McNemar's statistical test to compare the utilization of GDMT at baseline and after program optimization. Based on relevant literature [6] and clinical judgment, we categorized the four most common AEs (hypotension, bradycardia, hyperkalemia, and renal dysfunction) into three levels: mild, moderate, and severe (Table 1). Patients who experienced an AE but were later able to be rechallenged and uptitrated successfully on the associated therapy were not considered to have experienced an AE for the purposes of this study, as our goal was to identify AEs that truly prevented GDMT optimization. The frequency of each dose‐limiting AE was obtained for statistical analyses. In cases where patients reported multiple AEs, they were categorized based on the highest level experienced. Baseline patient characteristics, such as demographics, HF onset and etiology, laboratory results, and comorbidities, were collected and analyzed. The means and standard deviations are provided for continuous variables, and percentages are shown for categorical variables. Logistic regression models were developed to evaluate the association between patient factors and the titration to < 50% of the target dose for BBs and ACEis/ARBs/ARNIs, as these drug classes were the most frequently suboptimally dosed within our patient cohort. Variables with a *p* value ≤ 0.10 in the univariate model were included in the multivariate analysis. Additionally, the Charlson Comorbidity Index (CCI), which predicts survival in patients with multiple comorbidities, and the MAGGIC score, a risk stratification tool for mortality in HF, were calculated for each patient [13, 14]. Univariate regression analyses were performed to describe the association between these scores and achieving GDMT target doses. All tests were 2‐sided, and *P*‐values < 0.05 were considered statistically significant. All statistical analyses were performed with EZR Version 1.65.

**Table 1:** Grading of severity of GDMT‐related adverse effects.

	Hypotension	Bradycardia	Hyperkalemia	Renal dysfunction
Mild	Asymptomatic + SBP < 100 mmHg	Asymptomatic + < 60 bpm	5.1–5.4 mmol/L	25%–50% increase serum creatinine or 10%–25% decrease eGFR
Moderate	Symptomatic + SBP 90–100 mmHg	Symptomatic + 50–60 bpm	5.5–5.9 mmol/L	50%–100% increase serum creatinine or 25%–50% decrease eGFR
Severe	Symptomatic + SBP < 90 mmHg	Symptomatic + < 50 bpm	≥ 6.0 mmol/L	≥ 100% increase serum creatinine or ≥ 50% decrease eGFR

Abbreviations: bpm, beats per minute; eGFR, estimated glomerular filtration rate; GDMT, guideline‐directed medical therapy; SBP, systolic blood pressure.

## 3 Results

### 3.1 Patient Characteristics

Among the 327 patients enrolled in the program, 254 successfully completed the GDMT optimization program, and their baseline characteristics are shown in Table 2. The mean age at enrollment was 64 years old, 69.7% were male, and 80.3% were white. Sixty‐five percent had nonischemic HF, 53.5% had new‐onset (< 6 months) HFrEF or HFmrEF, and 43.7% had a recent HF hospitalization. The mean ejection fraction was 30.3%. Comorbidities included atrial fibrillation (32.7%), coronary artery disease (35.8%), and hypertension (65.4%). Forty‐eight (18.9%) patients achieved target GDMT doses, while 206 (81.1%) achieved the maximum tolerated dose of GDMT. Patients who achieved target doses were younger (mean [SD] age, 59 [13.4] years vs. 65 [13.02] years; *p* < 0.01), more likely to have nonischemic HF (39 [81.2%] vs. 126 [61.2%]; *p* = 0.017), less likely to have a recent HF‐related hospitalization (14 [29.2%] vs. 97 [47.1%]; *p* = 0.025), had less coronary artery disease (9 [18.8%] vs. 82 [39.8%]; *p* < 0.01), and were more likely to be obese (17 [35.4%] vs. 32 [15.5%]; *p* < 0.01). The two subgroups also differed significantly in racial demographics (*p* = 0.01).

**Table 2:** Baseline patient characteristics grouped by those who achieved maximum tolerated versus target GDMT doses.

Characteristic		All patients *n* = 254	Max tolerated *n* = 206	Target *n* = 48	*P* value
Demographics					
Age, mean (SD), years		64.29 (13.30)	65.48 (13.02)	59.19 (13.40)	**0.003**
Male sex (%)		177 (69.7)	143 (69.4)	34 (70.8)	1
Race (%)	Asian	10 (3.9)	9 (4.4)	1 (2.1)	**0.011**
	Black	22 (8.7)	11 (5.3)	11 (22.9)	
	Hispanic or Latino	10 (3.9)	8 (3.9)	2 (4.2)	
	White	204 (80.3)	170 (82.5)	34 (70.8)	
	Two or more races	2 (0.8)	6 (2.9)	0 (0.0)	
	Unknown	6 (2.4)	2 (1.0)	0 (0.0)	
Etiology (%)	Ischemic	88 (34.6)	79 (38.3)	9 (18.8)	**0.017**
	Nonischemic	165 (65.0)	126 (61.2)	39 (81.2)	
	Unknown	1 (0.4)	1 (0.5)	0 (0.0)	
HF onset (%)	Chronic	118 (46.5)	92 (44.7)	26 (54.2)	0.263
	New	136 (53.5)	114 (55.3)	22 (45.8)	
NYHA class (%)	I	7 (2.8)	5 (2.4)	2 (4.2)	0.711
	II	128 (50.4)	105 (51.0)	23 (47.9)	
	III	119 (46.9)	96 (46.6)	23 (47.9)	
Recent HF hospitalization (%)	No	143 (56.3)	109 (52.9)	34 (70.8)	**0.025**
	Yes	111 (43.7)	97 (47.1)	14 (29.2)	
Ejection fraction, mean (SD), %		30.32 (8.48)	30.30 (8.33)	30.38 (9.19)	0.958
Laboratory results, mean (SD)					
Creatinine, mg/dL		1.15 (0.36)	1.15 (0.37)	1.15 (0.31)	0.909
NT‐proBNP, pg/mL		2760.92 (4359.67)	2833.76 (4471.94)	2436.42 (3865.74)	0.637
Potassium, mEq/L		4.31 (0.45)	4.32 (0.44)	4.24 (0.49)	0.226
Comorbidities					
Atrial fibrillation (%)		83 (32.7)	71 (34.5)	12 (25.0)	0.235
Chronic kidney disease (%)		32 (12.6)	26 (12.6)	6 (12.5)	1
Coronary artery disease (%)		91 (35.8)	82 (39.8)	9 (18.8)	**0.007**
Hypertension (%)		166 (65.4)	133 (64.6)	33 (68.8)	0.618
Obesity (%)		49 (19.3)	32 (15.5)	17 (35.4)	**0.004**
Obstructive sleep apnea (%)		49 (19.3)	39 (18.9)	10 (20.8)	0.839

*Note:* Bold values indicate statistically significant at *p* < 0.05.

Abbreviations: GDMT, guideline‐directed medical therapy; HF, heart failure; NT‐proBNP, N‐terminal pro b‐type natriuretic peptide; NYHA, New York Heart Association.

### 3.2 GDMT Utilization Rates Before Versus After Program

**Figure 1:**
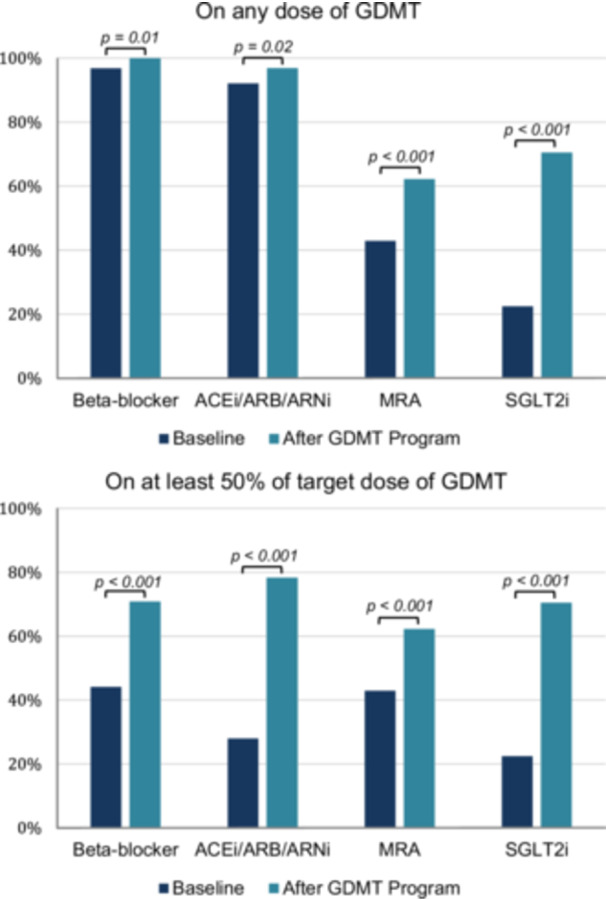
Changes in GDMT utilization from baseline to post‐program optimization by drug class. The figure illustrates changes in the use of any dose of GDMT and at least 50% of the target GDMT dose.

The change in the use of GDMT from baseline to post‐program optimization, both for any amount and for at least 50% of the target dose in each drug class, is shown in Figure 1. For BBs, the percentage of patients on any amount increased from baseline to after optimization (246 [96.9%] vs 254 [100%]; *p* = 0.01). The percentage of patients on at least 50% of the BB target dose also increased from 112 (44.1%) to 180 (70.9%) after program optimization (*p* < 0.001). For ACEis/ARBs/ARNIs, those on any amount increased from baseline (234 [92.1%] vs. 246 [96.9%]; *p* = 0.02). The percentage of patients on at least 50% of the ACEi/ARB/ARNI target doses increased from 71 (28.0%) to 199 (78.4%) after optimization (*p* < 0.001). For mineralocorticoid receptor antagonists (MRA), the percentage of patients on any amount and on at least 50% of the target dose increased from baseline to after optimization (109 [42.9%] vs. 158 [62.2%]; *p* < 0.001). For SGLT2 inhibitors, the percentage of patients on any amount and on at least 50% of the target dose increased from baseline to after optimization (57 [22.4%] vs. 179 [70.5%]; *p* < 0.001).

### 3.3 Adverse Effects

**Figure 2:**
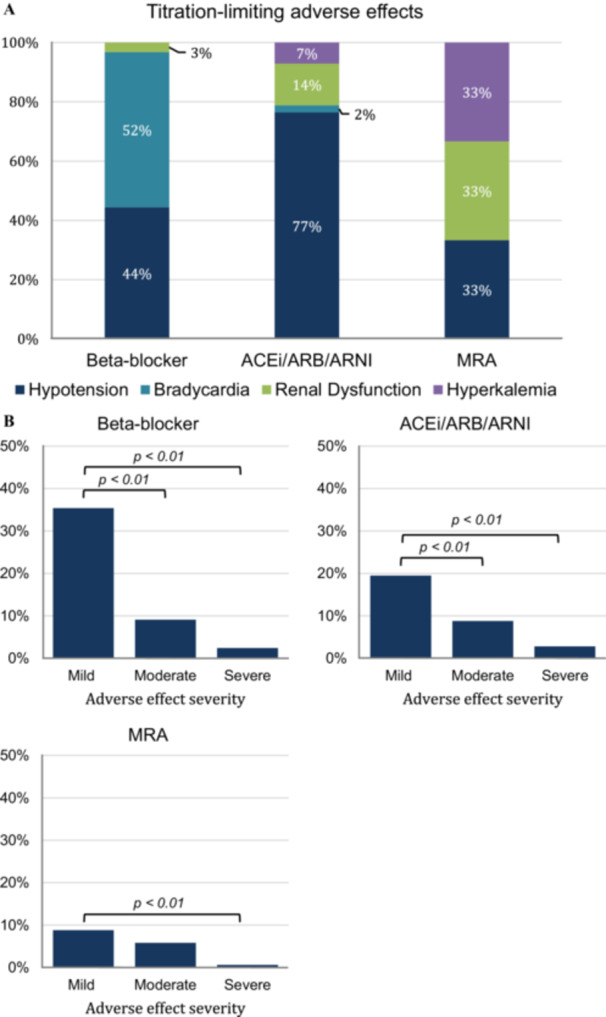
Adverse effects. (A) Distribution of titration‐limiting adverse effects by GDMT drug class, (B) Proportion of categorized adverse effects from beta‐blockers, ACE inhibitors/ARBs/ARNIs, and MRAs.

Of the patients who completed the program, 150 (59.1%) encountered at least one AE that hindered the titration to target GDMT doses. Figure 2A shows the distribution of the titration‐limiting AEs for each GDMT drug class. Among AEs experienced due to a BB, 66 (52.4%) was bradycardia, 56 (44.4%) hypotension, and 4 (3.2%) renal dysfunction. Among AEs related to an ACEi/ARB/ARNI, 65 (76.5%) was hypotension, 12 (14.1%) renal dysfunction, 6 (7.1%) hyperkalemia, and 2 (2.4%) bradycardia. The AEs from an MRA included hyperkalemia (*n* = 10; 33.3%), renal dysfunction (*n* = 10; 33.3%), and hypotension (*n* = 10; 33.3%). Lastly, among patients trialed on an SGLT2 inhibitor, none developed any of the four titration‐limiting AEs.

Figure 2B demonstrates the severity of AEs experienced due to each GDMT medication class. Of the patients who received a BB, 119 (46.9%) reported experiencing an AE, categorized as mild, moderate, or severe (35.4% vs. 9.1% vs. 2.4%; *p* < 0.01). Among patients receiving an ACEi/ARB/ARNI, 78 (31.1%) reported an AE, categorized as mild, moderate, or severe (19.5% vs. 8.8% vs. 2.8%; *p* < 0.01). Of the patients trialed on an MRA, 26 (15.2%) reported an AE, categorized as mild, moderate, or severe (8.8% vs 5.8% vs 0.6%; *p* < 0.01).

### 3.4 Achieving Target Dose Versus Maximum Tolerated Dose

**Figure 3:**
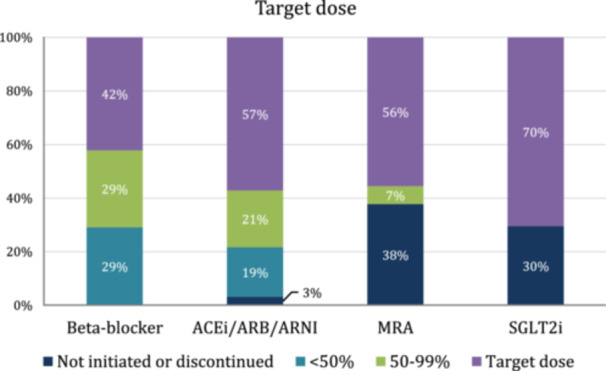
Proportion of patients achieving < 50%, 50%–99%, and target dose by GDMT drug class.

The percentages of subjects who achieved the target dose for each class of GDMT are shown in Figure 3. All patients who completed the program were on a BB with 107 (42.1%) achieving the target dose, 73 (28.7%) achieving 50%–99% target dose, and 74 (29.1%) achieving less than 50% of the target dose. For ACEis/ARBs/ARNIs, 145 (57.1%) patients reached the target dose, 54 (21.3%) reached 50%–99% of the target dose, 47 (18.5%) reached less than 50% of the target dose, and 8 (3.1%) patients were not initiated or discontinued on an ACEi/ARB/ARNI. For MRA therapy, 141 (55.5%) reached target doses, 17 (6.7%) reached 50%–99% target dose, and 96 (37.8%) were not initiated or discontinued on an MRA. For SGLT2 inhibitors, 179 (70.5%) of patients achieved the target dose and 75 (29.5%) were not started or discontinued on an SGLT2 inhibitor.

Submaximal dosing was more prevalent for BBs and ACEis/ARBs/ARNIs. Predictors of submaximal dosing for these drug classes were further analyzed through logistic regression models as shown in Tables 3A and 3B. Univariate analyses showed associations between BB suboptimal dosing (< 50% target dose) and older age, higher EF, ischemic etiology of HF, the presence of atrial fibrillation and coronary artery disease, and the absence of obesity. Multivariate analyses revealed significant associations between BB suboptimal dosing and older age (odds ratio [OR]: 1.04; 95% confidence interval [CI]: 1.0–1.07; *p* = 0.031), presence of atrial fibrillation (OR: 2.57; 95% CI: 1.18–5.58; *p* = 0.017), and absence of hypertension (OR: 0.39; 95% CI: 0.17–0.89; *p* = 0.025). Although strong factors in the univariate model, EF, ischemic etiology, coronary artery disease, and obesity lost significance in multivariate analysis. Univariate analyses showed associations between ACEi/ARB/ARNI suboptimal dosing (<50% target dose) and older age, ischemic etiology, higher creatinine levels before enrollment, presence of atrial fibrillation, and absence of obesity. Multivariate analyses revealed significant associations between ACEi/ARB/ARNI suboptimal dosing and presence of atrial fibrillation (OR: 2.08; 95% CI: 1.04–4.17; *p* = 0.039) while age, ischemic etiology, creatinine level, and obesity lost significance in multivariate analysis.

**Table 3A:** Logistic regression model of association between patient factors and beta‐blocker suboptimal dosing (< 50% target dose).

Factors	Univariate		Multivariate	
	Odds ratio (95% CI)	*p* value	Odds ratio (95% CI)	*p* value
Age	1.04 (1.02–1.07)	**0.00042**	1.04 (1.00–1.07)	**0.031**
Male sex	0.73 (0.41–1.30)	0.28	0.73 (0.31–1.72)	0.47
EF	1.04 (1.01–1.08)	**0.019**	1.03 (0.98–1.08)	0.26
NYHA class III or IV	1.03 (0.60–1.76)	0.93		
New onset HF	1.11 (0.65–1.91)	0.7		
Nonischemic etiology	0.53 (0.31–0.93)	**0.028**	0.97 (0.31–3.05)	0.95
Creatinine before enrollment	1.03 (0.48–2.19)	0.94		
NT‐proBNP before enrollment	1.00 (1.00–1.00)	0.19	1.00 (1.00–1.00)	0.75
Potassium level before enrollment	0.74 (0.40–1.36)	0.33		
Atrial fibrillation	2.47 (1.40–4.34)	**0.0017**	2.57 (1.18–5.58)	**0.017**
Chronic kidney disease	0.79 (0.34–1.84)	0.58	0.52 (0.16–1.71)	0.28
Coronary artery disease	1.99 (1.14–3.46)	**0.015**	2.31 (0.75–7.13)	0.14
Hypertension	0.64 (0.37–1.12)	0.12	0.39 (0.17–0.89)	**0.025**
Obesity	0.41 (0.18–0.93)	**0.032**	0.62 (0.18–2.09)	0.44
Obstructive sleep apnea	1.23 (0.63–2.40)	0.55		

*Note:* Bold values indicate statistically significant at *p* < 0.05.

Abbreviations: EF, ejection fraction; HF, heart failure; NT‐proBNP, N‐terminal pro b‐type natriuretic peptide; NYHA, New York Heart Association.

**Table 3B:** Logistic regression model of association between patient factors and ACEi/ARB/ARNI suboptimal dosing (< 50% target dose).

	Univariate		Multivariate	
Factors	Odds ratio (95% CI)	*p* value	Odds ratio (95% CI)	*p* value
Age	1.03 (1.01–1.06)	**0.0089**	1.02 (0.99–1.04)	0.3
Male sex	0.80 (0.42–1.52)	0.49	0.63 (0.30–1.34)	0.23
EF	1.00 (0.97–1.04)	0.88		
NYHA class III or IV	1.41 (0.77–2.61)	0.27		
New onset HF	1.22 (0.66–2.26)	0.53		
Nonischemic etiology	0.53 (0.28–0.99)	**0.047**	0.60 (0.22–1.64)	0.32
Creatinine before enrollment	2.60 (1.07–6.31)	**0.035**	2.56 (0.95–6.88)	0.062
NT‐proBNP before enrollment	1.00 (1.00–1.00)	0.3		
Potassium level before enrollment	1.38 (0.69–2.76)	0.36		
Atrial fibrillation	2.36 (1.26–4.42)	**0.007**	2.08 (1.04–4.17)	**0.039**
Chronic kidney disease	1.39 (0.58–3.32)	0.46		
Coronary artery disease	1.81 (0.97–3.36)	0.062	1.00 (0.37–2.70)	0.99
Hypertension	0.75 (0.40–1.40)	0.37		
Obesity	0.38 (0.14–1.00)	**0.05**	0.50 (0.18–1.41)	0.19
Obstructive sleep apnea	0.47 (0.19–1.18)	0.11	0.50 (0.18–1.38)	0.18

*Note:* Bold values indicate statistically significant at *p* < 0.05.

Abbreviations: EF, ejection fraction; HF, heart failure; NT‐proBNP, N‐terminal pro b‐type natriuretic peptide; NYHA, New York Heart Association.

Univariate regression analyses evaluating the association between CCI and MAGGIC scores and achieving target doses of GDMT were conducted. These studies showed that higher CCI scores are less likely to reach GDMT target doses (OR: 0.68; 95% CI: 0.56–0.82; *p* < 0.01). Similarly, higher MAGGIC risk scores are less likely to reach GDMT target doses (OR: 0.93; 95% CI: 0.89–0.98; *p* < 0.01).

## 4 Discussion

Our study measured the frequency and severity of adverse effects experienced by patients in a specialized GDMT optimization program. Patients on BBs most commonly experienced bradycardia, while patients on ACEis/ARBs/ARNIs most commonly experienced hypotension. The AEs from MRAs were equally distributed between hyperkalemia, renal dysfunction, and hypotension. Adverse effects that restricted the uptitration of these GDMT drug classes within our program were predominantly mild and asymptomatic. These findings align with a study that analyzed rates of AEs across 17 landmark clinical trials on GDMT for HFrEF, where only 4.7% to 13.4% of patients discontinued study drug due to AEs [15]. However, the lack of a unified definition of intolerance to GDMT across the clinical trials highlights the need for a standardized way of titrating or rechallenging GDMT for patients who develop AEs.

Previous studies have shown that frail patients are less likely to receive optimal GDMT therapy [16, 17]. In a post hoc analysis of the GUIDE‐IT trial, 56% of participants with HFrEF had a high frailty burden at baseline, were older, had higher BMIs, NT‐proBNP levels, and number of comorbidities. This frailty burden was associated with a lower probability of being initiated on and uptitrated to optimal GDMT which was independently associated with a higher risk of adverse clinical outcomes [16]. Interestingly, a study from the Swedish Heart Failure Registry found that obese patients were more likely to receive GDMT and achieve target doses even after adjusting for characteristics linked to tolerability, suggesting that perceived, rather than actual physiological limitations, may influence undertreating of nonobese patients [18]. Similarly, our patient cohort revealed that younger age and having obesity at baseline were associated with a higher likelihood of achieving target doses of GDMT, while older age was a significant predictor of suboptimal dosing of BBs and ACEis/ARBs/ARNIs. Additionally, patients with higher CCI and MAGGIC HF scores were less likely to achieve target doses. This may reflect that both underlying frailty and perceived tolerance contributed to the challenges in optimizing GDMT therapy for certain patients, consistent with the findings of the above‐mentioned studies.

Therapeutic inertia, the failure to intensify therapy when indicated, in clinical practice is often described as a significant contributing factor to suboptimal GDMT therapy [19, 20, 21]. In a secondary analysis of the GUIDE‐IT trial, clinicians citing patients as “clinically stable” or “already at maximally tolerated therapy” were the most common reasons for not adjusting GDMT doses. Furthermore, most GDMT adjustments were made within the first 6 weeks of the trial with a significant decline in medication titration after this period, suggesting a reluctance to further adjust therapy once patients achieve apparent clinical stability. Even in cases of clinical stability, however, elevated NT‐proBNP levels indicated opportunities for medication optimization [22]. Actual or fear of adverse effects can serve as a clinical barrier to GDMT optimization, and the response to these concerns often varies among clinicians. In a study that surveyed physicians, primarily cardiologists, 54% indicated that they would reduce or discontinue MRA treatment in patients with or at risk of hyperkalemia while only 18% would opt for a novel potassium binder, despite evidence supporting their use [23]. Conversely, other studies suggest that the effect of clinical inertia on suboptimal GDMT use may be overestimated as physiological limitations pose a bigger challenge [24, 25]. In the present study, it remains unclear whether the underutilization of GDMT in certain patients is primarily driven by clinical inertia and perceived tolerance or actual physiological limitations. While one might expect that titration‐limiting AEs within our protocol‐driven program, led by a highly specialized HF team, would be more severe given the presence of patient factors related to frailty and tolerability, the generally mild and asymptomatic AEs limiting medication titration highlight the complexity of optimizing HFrEF treatment. This demonstrates the need for improved patient engagement in discussions about the risks associated with not escalating GDMT [26]. Additionally, others have emphasized the importance of setting realistic goals for the proportion of patients expected to be on target doses, as achieving the target dose may not be feasible for every patient [27].

### 4.1 Study Limitations

This was a single‐center, observational study with a relatively small sample size, which may limit the generalizability of our findings to other settings. Adverse effects were analyzed retrospectively, which may introduce recall bias and affect the accuracy of AE categorization. Additionally, although medications were titrated sequentially, it was not always possible to attribute each adverse effect to one specific medication. Lastly, our patient cohort included participants who underwent titration before guidelines were updated to recommend SGLT2 inhibitors.

### 4.2 Conclusions

Our study identified key patient characteristics that influence the achievement of target GDMT doses and described the frequency and severity of AEs that limited further titration of medications. In general, our multidisciplinary and telehealth‐based program achieved significantly higher GDMT utilization rates. However, many participants with certain characteristics, such as older age, and comorbidities, including atrial fibrillation and coronary artery disease, did not reach target GDMT doses, and most adverse effects to the medications were mild and asymptomatic. These findings suggest that the relative contributions of clinical inertia and physiological limitations are complex, and GDMT optimization may not be a one‐size‐fits‐all approach. Further studies are needed to standardize adverse effects definitions and management of GDMT to better understand and address implementation barriers.

## Conflicts of Interest

The authors declare no conflicts of interest.

## Data Availability

The datasets generated during and/or analyzed during the current study are available from the corresponding author on reasonable request.
